# CO_2_-Laser-Micromachined, Polymer Microchannels with a Degassed PDMS slab for the Automatic Production of Monodispersed Water-in-Oil Droplets

**DOI:** 10.3390/mi13091389

**Published:** 2022-08-25

**Authors:** Akitsu Ogo, Shotaro Okayama, Masaya Nakatani, Masahiko Hashimoto

**Affiliations:** Department of Chemical Engineering and Materials Science, Faculty of Science and Engineering, Doshisha University, 1-3 Tataramiyakodani, Kyotanabe 610-0321, Japan

**Keywords:** droplet, microchannel, CO_2_-laser machining, polydimethylsiloxane (PDMS), poly(methyl methacrylate) (PMMA)

## Abstract

In our recent study, we fabricated a pump/tube-connection-free microchip comprising top and bottom polydimethylsiloxane (PDMS) slabs to produce monodispersed water-in-oil droplets in a fully automated, fluid-manipulation fashion. All microstructures required for droplet production were directly patterned on the surfaces of the two PDMS slabs through CO_2_-laser micromachining, facilitating the fast fabrication of the droplet-production microchips. In the current extension study, we replaced the bottom PDMS slab, which served as a microfluidic layer in the microchip, with a poly(methyl methacrylate) (PMMA) slab. This modification was based on our idea that the bottom PDMS slab does not contribute to the automatic fluid manipulation and that replacing the bottom PDMS slab with a more affordable and accessible, ready-to-use polymer slab, such as a PMMA, would further facilitate the rapid and low-cost fabrication of the connection-free microchips. Using a new PMMA/PDMS microchip, we produced water-in-oil droplets with high degree of size-uniformity (a coefficient of variation for droplet diameters of <5%) without a decrease in the droplet production rate (~270 droplets/s) as compared with that achieved via the previous PDMS/PDMS microchip (~220 droplets/s).

## 1. Introduction

Monodispersed water-in-oil (W/O) droplets can serve as uniformly sized, discrete compartments for individual molecules or cells and allow for highly parallel processing within the droplets. Because of these distinguishing characteristics, monodispersed W/O droplets have been increasingly used in various cutting-edge biochemical studies on droplet-based polymerase chain reactions [1,2], single-cell deoxyribonucleic acid sequencing [3,4], single-cell sorting [5,6], enzyme-linked immunosorbent assays [7,8], directed evolution [9,10], protein crystallization [11], and drug screening [12,13].

To date, it appears that the most promising way to produce monodispersed W/O droplets is to use a microfluidic chip in which an oil and aqueous solution, flowing in separate microfluidic channels, merge at a channel junction (e.g., adopting a T-junction [14,15] or flow focusing [16,17]) to form discontinuous, aqueous droplets in a continuous oil flow. For microfluidic droplet production, a microfluidic system generally requires external pumping devices, such as syringe pumps, to deliver an oil phase and an aqueous phase into the microchannels of a chip. Such microfluidic systems allow for the production of monodispersed W/O droplets at appreciable rates (to a few kHz [18,19]), and the droplet size can be changed by adjusting the flow-rate ratio of the oil and aqueous phases. However, the use of external pumping devices tends to make a microfluidic droplet production system costly, bulky, and complex.

Hosokawa et al. established a unique technique that allows for the automatic liquid transport in microfluidic channels fabricated on a polydimethylsiloxane (PDMS) microchip without the use of external pumping devices [20]. This technique takes advantage of the high gas permeability of PDMS, and the principle of the automatic liquid transport is as follows. Air dissolved in a bulk PDMS microchip can be easily evacuated under a vacuum environment. When the degassed PDMS microchip is returned to atmospheric pressure, air automatically starts to dissolve into the chip. If a liquid is loaded into the inlet end of a microchannel with the outlet space of the microchannel sealed, the liquid flows to the outlet space because a reduced pressure is generated in the outlet void space, owing to the dissolution of the air entrapped in that space into the PDMS substrate (see Figure S1 for a schematic representation of the principle). We used this automatic liquid transport method with a degassed PDMS microfluidic chip free of a pump/tube connection (hereafter referred to as a connection-free microchip) for the automated production of W/O droplets with high size-uniformity (a coefficient of variation (CV) for droplet diameters of <3%) [21]. Our method of droplet production has three major drawbacks. (1) The droplet size is predetermined by the microchannel dimensions for a given water/oil phase combination. Therefore, to produce droplets of an intended size, a new microchip with different microchannel dimensions needs to be designed and fabricated. (2) The run duration for droplet production with the chip is limited by the liquid volume filling the outlet space. (3) The droplet-generation rate is limited by the level of the pressure drop due to the permeation of air into the PDMS. Despite these limitations, our droplet production method only requires the degassing of a PDMS microfluidic chip prior to the injection of the oil phase and aqueous phase into separate inlet reservoirs, offering a compact, easy-to-use, and convenient automated system for the microfluidic production of monodispersed W/O droplets.

In addition to the facile operational procedures, another advantage of our droplet-production method adopting the automatic liquid-transport approach is that, owing to the manner of the vacuum-driven fluid manipulation, the PDMS sheet possessing the microchannels does not need to be irreversibly bound to a PDMS or glass cover-plate using an elaborate technique (e.g., oxygen plasma treatment for irreversible PDMS/PDMS or PDMS/glass bonding [22,23]) to avoid liquid leakage from the microchannels. In fact, we confirmed in our previous study that fluids did not leak from the microchannels during runs with the PDMS microfluidic sheet reversibly adhered to a cover-plate through the viscoelastic properties of PDMS [24]. This contrasts with the conventional microfluidic droplet-production approach in which liquids are pressurized to deliver fluids into microchannels, inevitably resulting in liquid leakage from the microchannels if the microchannels are not sealed with a cover-plate completely. Elimination of the complete enclosure of microchannels with a cover-plate greatly simplifies the fabrication of our microfluidic droplet-production chips. However, to make the microfluidic channels, we adopted conventional soft lithography, which required many manufacturing processes with expensive requisites (e.g., elaborate photomasks, a spin-coater, and an ultraviolet exposure apparatus). Therefore, the fabrication of the chips remained slow and costly.

We recently adopted CO_2_-laser micromachining, which is simpler, faster, and less expensive than standard soft lithography [25,26,27,28,29,30,31,32,33,34,35], to rapidly fabricate a connection-free PDMS microfluidic chip for the automatic production of monodispersed W/O droplets [36]. This chip comprises top and bottom PDMS slabs that are reversibly bonded together. The bottom PDMS slab has laser-engraved microchannels for two liquids (i.e., an oil phase and aqueous phase) to flow, while the top PDMS slab seals the microchannels. In view of the liquid-suctioning mechanism for this reversibly assembled PDMS/PDMS microchip, however, we consider that the material of choice for the bottom slab as a microfluidic layer should not be limited to PDMS, but that polymer materials that are more accessible and affordable than PDMS could possibly be adopted because only the top PDMS slab appears to serve as a liquid-suctioning actuator although the bottom PDMS slab is also degassed together with the top PDMS slab in the PDMS/PDMS microchip assembly, and a pristine PDMS slab can be reversibly adhered to the smooth surfaces of many other polymer materials according to the viscoelastic properties.

Poly(methyl methacrylate) (PMMA) is an accessible and affordable polymer with high levels of transparency, durability, and biocompatibility. In addition, many PMMA/PMMA bonding methods (such as thermal bonding [37,38], solvent bonding [39], and adhesive bonding [40]) have been established for tightly sealing microchannels. Therefore, in studies of microfluidic droplet-production that conducted direct laser-machining to engrave microchannels on the surface of a planar polymer substrate [25,26,27,28,29], PMMA has most frequently been chosen as the material to be processed. In the present study, we created a reversibly bonded, two-layer, polymer microchip comprising a bottom PMMA slab as a microfluidic layer and a top PDMS slab as a liquid-suction actuator for the production of monodispersed W/O droplets in a fully automated fashion of fluid manipulation. The choice of a commercially available, ready-to-use PMMA slab to be processed through direct laser-writing for microchannel fabrication further facilitated the rapid and low-cost fabrication of the connection-free microfluidic droplet-generator. One of our major aims in this extension study is to investigate whether the replacement of the bottom PDMS microfluidic layer with a different polymer substrate affects the basic performance of the connection-free microfluidic droplet-generator (mainly the droplet production rate). We also describe what is likely the most critical factor that determines the droplet-production rate for our connection-free microchips.

We found in the current study that the highest volumetric-flow rate of microchannels attainable with the automatic liquid-delivery method is not affected by the solid volume of the degassed top PDMS slab as a sole and consistent vacuum source of the microchip, while the rate is likely to be affected by the adhesion tightness between the top, viscoelastic PDMS slab and a bottom, polymer slab that should be determined inherently by the surface properties (e.g., hydrophobicity and hydrogen bonding) of the bottom material. The adoption of the automatic liquid-delivery method using a degassed PDMS slab is not limited to microfluidic droplet-creation, but rather it is suitable for many other applications, such as nucleic-acid detection [41,42,43,44], immunoassays [45,46], mercury detection [47], and viscosity measurements [48,49]. Therefore, this finding suggests that the established applications can be performed using the liquid-delivery method with a reduced size of the PDMS microchip without reducing the volumetric-flow rates of the microchannels.

## 2. Materials and Methods

**Figure 1:**
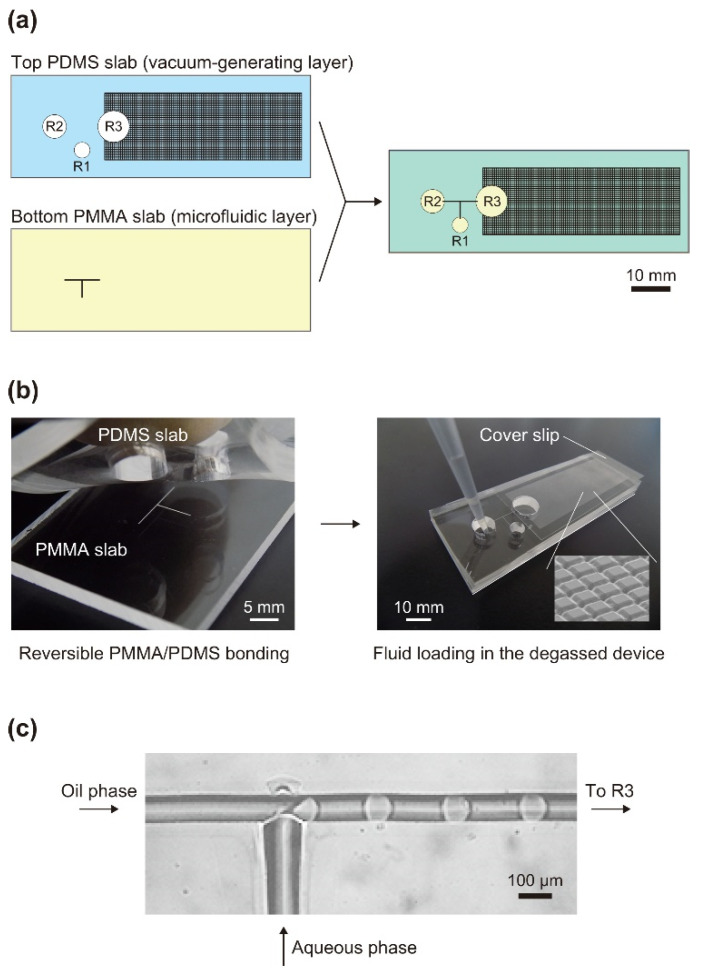
Schematics of the polymer microfluidic chip: (**a**) Drawings showing the chip structure. The shaded rectangular area on the top surface of the top polydimethylsiloxane (PDMS) slab indicates a region of arrayed, orthogonal microgrooves produced through direct-laser patterning. (**b**) Photographs of the chip. Inset: Scanning electron microscopy image of a laser-written microgroove array. (**c**) Optical microscopy image of immiscible flows in a channel in the vicinity of a T-junction.

A polymer microfluidic chip with a horizontal area similar to that of a standard microscope slide (76 mm × 26 mm) was designed and tested in the current study (Figure 1a). The microchip comprised a PMMA slab (a bottom layer) and PDMS slab (a top layer), having thicknesses of 2 and 4 mm, respectively. The bottom PMMA slab had T-junction microfluidic channels on the upper surface, which was patterned using a CO_2_-laser-engraving machine (ML-Z9550TA; KEYENCE, Osaka, Japan) that emitted infrared radiation at a wavelength of 9.3 µm. The output power of the laser can be changed relative to the maximum output power of 20 W. The size of the best-focused laser spot that the laser-engraving machine generates is 80 µm in diameter, and the smallest groove that can be fabricated with the machine is thus considered to be approximately 80 µm in width. The lengths of the 3 microfluidic paths from the T-junction point to each of the terminal points were all 4 mm. In the top PDMS slab, 3 through-holes (R1, R2, and R3) were cut. In addition, a two-dimensional array of orthogonal microgrooves (covering an area of 850 mm^2^) was fabricated on the upper surface of the top PDMS slab using the laser-engraving machine to increase the PDMS surface-area-to-volume (SA/V) ratio inside the air-trapping void space, facilitating the permeation of the entrapped air into the degassed PDMS slab, and thereby increasing the vacuum level in that space [50].

The bottom PMMA and top PDMS slabs were reversibly bonded together on the basis of the viscoelastic properties of PDMS (Figure 1b, left). The opening of the outlet reservoir (R3), which served as a droplet-collection space, was reversibly sealed with a 24 mm × 60 mm microscope coverslip (thickness of 0.13–0.17 mm). The PDMS chip was placed in a conventional vacuum desiccator and degassed at approximately 300 Pa for 1.5 h. After the chip was returned to atmospheric pressure, 25 µL of an aqueous phase (0.18% *w*/*v* indigo carmine; FUJIFILM Wako Pure Chemical Corporation, Osaka, Japan) was injected into inlet reservoir R1, and 50 µL of an oil phase (Novec 7500 containing 2% *w*/*w* Pico-Surf 1; Sphere Fluidics, Cambridge, UK) was injected into inlet reservoir R2. This operation resulted in the automatic generation of W/O droplets at the T-channel junction (Figure 1c). An inverted microscope (AXJ-5300TPHFL; Wraymer, Osaka, Japan) equipped with a high-speed camera (HAS-D71; DITECT, Tokyo, Japan) was used for the real-time monitoring of droplet generation. In the current study, the microchip shown in Figure 1 was used as the standard microchip, among other variant microchips. (See Table 1 and Figure S2.)

## 3. Results and Discussion

### 3.1. Laser Micromachining of Microfludic Channels on a PMMA Slab

Including the design and geometrical size, the standard model of the microfluidic chip used in the current study is identical to that used in our previous study [36], except for the polymer material deployed as a bottom microfluidic layer—namely, PMMA in the current study and PDMS in the preceding study. It is reasonable to consider that the droplet-production rate obtained with our preceding microchip (~220 droplets/s [36]) was determined by (1) the level of vacuum created within the microchip and (2) the hydrodynamic resistances specific to the given microchannels. This occurs because the vacuum environment created in the outlet space generates the fluid flows in the microchannels, and the rates of fluid flows must be determined by the vacuum level attained and the hydrodynamic resistances inherent to the given microchannels. On the basis of the structural architecture of the microchip, we hypothesize that the bottom PDMS microfluidic layer, which was degassed together with the top PDMS layer, did not contribute to the creation of the vacuum environment, and only the top PDMS layer likely creates the vacuum environment (as discussed in the following section). The material of choice for the bottom microfluidic substrate thus appears to bear no relation to the droplet-production rate. A comparison of the droplet-production rate between the preceding and current microchips, having identical top PDMS layers (and presumably the same attainable vacuum level), can test the validity of our hypothesis if the hydrodynamic resistances of the microchannels, which are determined by the geometrical patterns and dimensions (lengths and cross-sectional areas) of the microchannels, are the same for the two microchips. We therefore attempted to engrave the identical dimensions of microchannels with the T-junction channel pattern on the surfaces of a PMMA slab as those fabricated on PDMS surfaces in the preceding study in an effort to provide the same hydrodynamic resistances of the microchannels on the PMMA as those on the PDMS slab.

**Figure 2:**
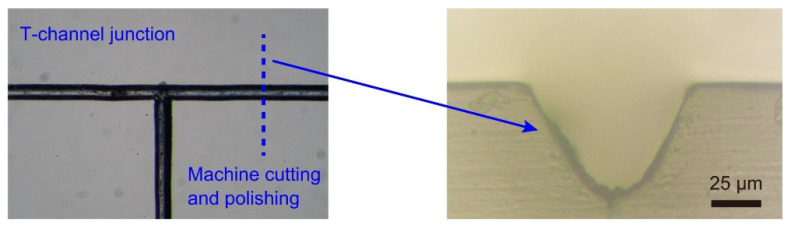
Microscopic observations of laser-engraved microfluidic channels: (**left**) top view of microchannels in the vicinity of a T-junction; (**right**) cross-sectional view of a microchannel.

Cross-sectional dimensions of 76-µm deep and 79-µm wide channels were obtained on surfaces of a PDMS slab under the given laser-writing conditions of the preceding study [36]. Various laser-writing parameters, such as the speed, power, focal distance, and number of passes, affect the cross-sectional size of a channel [26,28,29,30,31,32,33,34,35]. Through the management of the laser-writing conditions via a trial-and-error approach, we acquired T- junction channels with cross-sectional channel dimensions of 77 µm depth and 85 µm width on surfaces of a PMMA slab (Figure 2, right), which were similar to those on the PDMS surfaces.

### 3.2. Time-Course Measurements of Droplet-Production Rates Achieved with the Standard PMMA/PDMS Microchip

**Figure 3:**
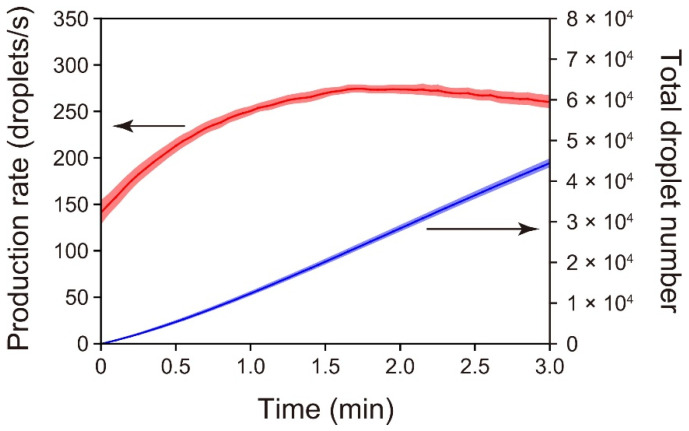
Changes in the rate of droplet-production over the time-course of the experiment (*n* = 5) for the PMMA/PDMS microchip. The red line shows the mean droplet-production rate. The blue line shows the mean total number of droplets produced, which was calculated by integrating the production rate with respect to time. The colored, shaded areas above and below the lines represent ±1 standard error of the mean.

We performed time-course measurements of the droplet-production rates using the standard PMMA/PDMS microchip (Figure 3). The moment at which droplet-production was initiated was set as time zero. The droplet-production rate initially increased and then slightly decreased. This characteristic of the rate change was also evident for the PDMS/PDMS chip used in the latest study [36] and our other preceding formats of connection-free droplet production chips [24,50,51]. The mechanism of the rate change was discussed in the cited studies in terms of the change in the vacuum level within the sealed, air-trapping outlet void space. Briefly, the pressure within the enclosed outlet space continues to decrease in the initial period of a run because the air entrapped in that space permeates into the air-evacuated PDMS slab after the inlet reservoirs are filled with liquid. However, the incoming liquid in the outlet reservoir counteracts the pressure decrease because the incoming liquid reduces the void volume in the outlet space, which eventually leads to an increasing trend of the pressure within the confined outlet.

The maximum droplet-formation rate attained with the present PMMA/PDMS chip (ca. 270 droplets/s) was a little higher than that attained with the former PDMS/PDMS chip (ca. 220 droplets/s) [36]. The increased droplet-production rate of the present PMMA/PDMS chip does not necessarily indicate larger volumetric-flow rates of the immiscible phases relative to those for the former PDMS/PDMS chip because the weighted mean diameter of 84 µm obtained in repeated runs with the PMMA/PDMS chip (shown below) is smaller than that obtained in runs with the PDMS/PDMS chip (94 µm) [36]. Based on movies of droplet-generation near the T-junction that were acquired under a microscope with a high-speed camera, the volumetric-flow rates of the aqueous and oil phases at the points where droplet-generation rates were maximized were estimated to be 84 and 110 nL/s, respectively, (194 nL/s in total) for the PMMA/PDMS microchip. Similarly, the volumetric-flow rates of the aqueous and oil phases were estimated to be 96 and 105 nL/s, respectively, (201 nL/s in total) for the PDMS/PDMS microchip. The hydrodynamic resistance of a parabolic channel is inversely proportional to *h*^3^*w* (where *h* is the shorter dimension, and *w* is the longer dimension). On the basis of the *h*^3^*w* values calculated from the individual *h* and *w* for the PMMA and PDMS microchannels (*h* = 77 µm and *w* = 85 µm for the PMMA microchannel; *h* = 76 µm and *w* = 79 µm for the PDMS microchannel), the hydrodynamic resistance of the PDMS microchannel relative to that of the PMMA microchannel was calculated to be 1.12, and the volumetric-flow rate of the PDMS microchannel was thus expected to be 1/1.12 times that of the PMMA microchannel (194 nL/s in total; see above); i.e., 173 nL/s in total. In contrast with this expectation, the actual volumetric-flow rate observed in the PDMS microchannel (201 nL/s) was 1.16 times the expected rate (173 nL/s). Although a PDMS slab appears to be able to reversibly adhere to the smooth surfaces of many materials owing to the viscoelastic property of PDMS, the degree of adhesion of PDMS may differ depending on the surface property (e.g., the hydrophobicity or hydrogen bonding) of the material to which it will adhere, and the tightness of the adhesion likely determines the vacuum level within the outlet space accordingly. We suggest that a PDMS slab cannot adhere to PMMA as strongly as it adheres to PDMS, resulting in a reduced vacuum level. This possibly explains why the lower volumetric-flow rate was observed for the PMMA/PDMS microchip, despite the lower hydrodynamic resistance, as compared with the PDMS/PDMS microchip. Whereas the difference in the volumetric-flow rate between the two microchips cannot simply be explained by the difference in the relative hydrodynamic resistance alone, we speculate that the bottom microfluidic PDMS slab of the PDMS/PDMS microchip, which was degassed together with the top PDMS slab, was unlikely able to serve as a consistent vacuum source during a run. The basis of this speculation is that the bottom surfaces of (1) the PDMS microfluidic paths and (2) the outlet reservoir (i.e., the top surfaces of the bottom PDMS slab at the relevant locations) were immediately wetted with the incoming fluids in the initial stage of the run (refer to Figure S1c,d), and the air entrapped inside the outlet space could not permeate the bottom PDMS slab. Thus, the top PDMS slab, on which most of the PDMS surfaces exposed to the entrapped air did not become wet with the flow of incoming liquid, served as a sole and consistent vacuum source for the chip, and the vacuum level attained inside the outlet space determined the flow rates of the immiscible fluids. In fact, the flow rates of the immiscible fluids were not appreciably different between the PMMA/PDMS and PDMS/PDMS microchips (having total volumetric-flow rates of 194 and 201 nL/s, respectively, as described above) although the material of the bottom microfluidic slab was different between the two microchips.

To evaluate the reproducibility of the automatic droplet-production with the PMMA/PDMS microchip, the above time-course measurements were repeated multiple times. Standard errors in the droplet-production rate and total droplet number, respectively, indicated in Figure 3 by the red- and blue-shaded regions around the curves, were sufficiently small throughout the droplet production. Specifically, the highest standard error in the droplet-production rate, appearing soon after the initiation of the droplet-production, was 9% of the mean rate. This value was higher than that obtained with the PDMS/PDMS microchip format in our preceding study (3%, *n* = 5) [36]. However, the value decreased as time passed. Specifically, 0.5 min after the initiation of the droplet production, the value remained below 3%.

As indicated by the blue line in Figure 3, the standard PMMA/PDMS microchip produced ca. 44,000 droplets with a diameter of 84 µm (shown later) in 3 min with an increasing trend in the droplet-generation rate (red line). This quantity and size of the droplets are useful in certain applications. For instance, the commercialized droplet digital PCR system (QX100) from Bio-Rad Laboratories Inc. prepares 20,000 droplets with a diameter of 124 µm [52]. However, there should be other applications that require the preparation of a much larger number of droplets with a reduced droplet size. As mentioned earlier, the size of the droplets that the current versions of a microchip with the simple T-channel junction can produce appears to be determined by the cross-sectional dimensions of the microchannels, which are limited by the spatial resolution specific to the laser-engraving machine. (One should recall that the size of the best-focused laser spot was 80 µm in diameter.) Therefore, it might be difficult to produce droplets with a diameter several times smaller than that of the droplets actually produced in the current study if the simple T-channel-junction geometry is adopted for droplet production. However, it may be possible to generate smaller droplets by configuring bifurcation junctions as droplet splitters downstream of a droplet-generating junction (e.g., T-junction or flow-focusing junction), as demonstrated by Hatch et al. [53]

**Figure 4:**
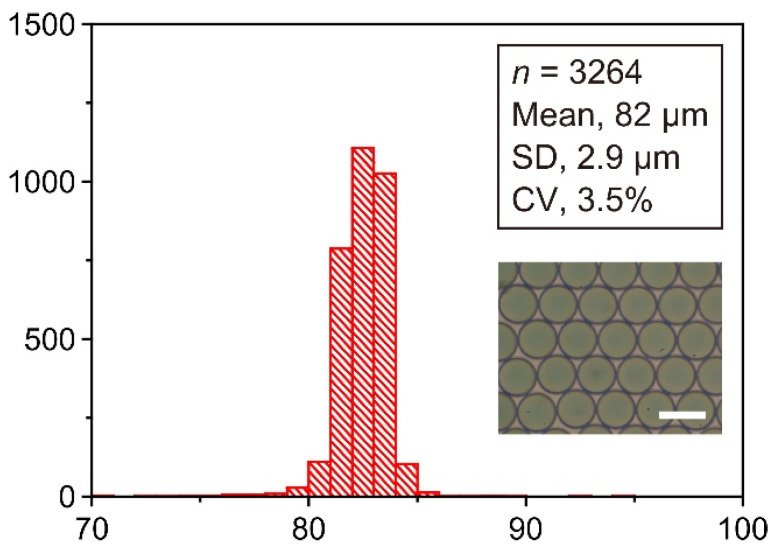
Diameter distribution of the analyzed droplets, showing the total droplet sample size (*n*), mean diameter, standard deviation (SD), and coefficient of variation (CV). Inset: Typical image of a droplet monolayer (scale bar, 100 µm). A standard PMMA/PDMS microchip was used in the experiments.

After the completion of the droplet-production, the droplets were retrieved from the outlet reservoir (R3 in Figure 1a), and their diameters were measured to evaluate their size uniformity. Figure 4 shows the size distribution of the droplets obtained in a single run. In that run, the mean droplet diameter was 82 µm, with a polydispersity of 3.5% (as indicated by the CV for the diameter). The polydispersity for droplets prepared with our preceding PDMS/PDMS microchip was 2.6% [36]. A comparable variation in the polydispersity was thus acquired with the current PMMA/PDMS microchip. We conducted the same experiment 5 times to evaluate the size consistency of the droplets produced in different runs (Table 2). The pooled standard deviation (SD) of the diameter, calculated from these repeated experiments, was 2.8 µm―corresponding to just 3.3% of the weighted mean diameter (84 µm) estimated from 5 distinct runs. The PMMA/PDMS microchip was thus confirmed to produce droplets with high level of size uniformity within each run and a good level of size reproducibility among different runs.

### 3.3. Droplet Production with a PET/PDMS Microchip

By conducting the experiments described in the previous section (Section 3.2), we showed that a bottom microfluidic layer was unlikely to contribute to the creation of a vacuum environment within a microchip even if a degassed PDMS slab was chosen for the bottom layer. However, to confirm whether the choice of polymer material for the bottom microfluidic layer affects the volumetric-flow rates of the immiscible fluids, we replaced the bottom PMMA slab of the standard PMMA/PDMS microchip with a poly(ethylene terephthalate) (PET) slab, which had microchannels with a cross-sectional area of 3540 µm^2^ (59 µm deep and 120 µm wide, with a Gaussian-like, cross-sectional profile) that was slightly larger than that of a standard microchip (3270 µm^2^), and we investigated whether this PET/PDMS microchip (Variant I in Table 1) provided volumetric-flow rates of the immiscible fluids comparable to those provided by the standard microchip (a total flow rate of the aqueous and oil phase of 194 nL/s, as described in Section 3.2).

**Figure 5:**
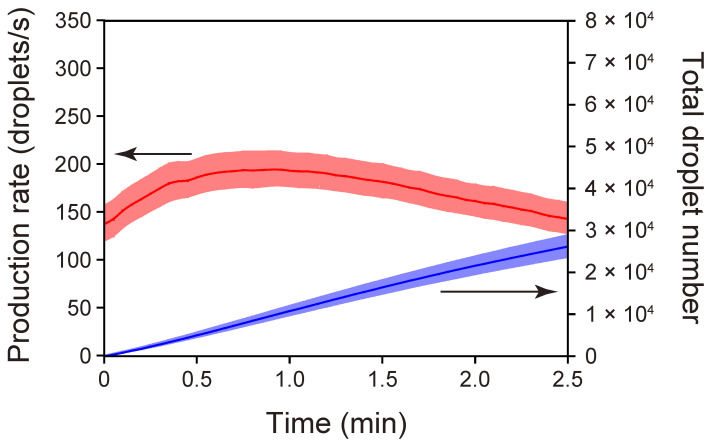
Time-course changes in the droplet-production rate and total droplet number for the PET/PDMS microchip (Variant I). The red line shows the mean droplet-production rate. The blue line shows the mean total number of droplets produced, which was calculated by integrating the production rate with respect to time. The colored, shaded areas above and below the lines represent ±1 standard error of the mean. The number of runs (*n*) = 3.

Similar to the experiments conducted using a standard PMMA/PDMS microchip, we performed time-course measurements of the droplet-production rate with the PET/PDMS microchip (Figure 5). Droplets with a diameter of 90 µm were obtained at a production rate of ~190 droplets/s, which was lower than that for the PMMA/PDMS microchip (ca. 270 droplets/s). As in the case of the analysis described in the previous section, we estimated the volumetric-flow rates of the aqueous and oil phases for the PET/PDMS microchip because the droplet-production rate does not directly represent the magnitude of the volumetric-flow rate. The measured volumetric-flow rates for the aqueous and oil phases were 76 and 107 nL/s, respectively (183 nL/s in total). This total volumetric flow was not largely different from that obtained with the PDMS/PDMS microchip (201 nL/s), confirming that the bottom microfluidic layer does not likely affect the vacuum level attained inside the outlet space—or, consequently, the volumetric-flow rate. In view of the hydrodynamic resistance of the PET microchannel relative to that of the standard PMMA microchannel (1.58), the expected total volumetric-flow rate of the PET microchannel should have been 1/1.58 times that of the PMMA microchannel (194 nL/s; see above)—namely 123 nL/s. Although the measured flow rate (183 nL/s) was not consistent with the expected flow rate (123 nL/s), the comparison of the volumetric-flow rate in terms of the hydrodynamic resistance based on the 1/*h*^3^*w* value well explains the reason that the observed volumetric-flow rate of the PET microchannel was (183 nL/s) rather smaller than the rates of the PDMS and PMMA microchannels (201 and 194 nL/s, respectively), despite the larger cross-sectional area of the PET microchannel (3540 µm^2^) as compared with those of the PDMS and PMMA microchannels (3000 and 3270 µm^2^, respectively).

### 3.4. Droplet-Production Using Different Versions of the PMMA/PDMS Microchip (Variant II and Variant III)

**Figure 6:**
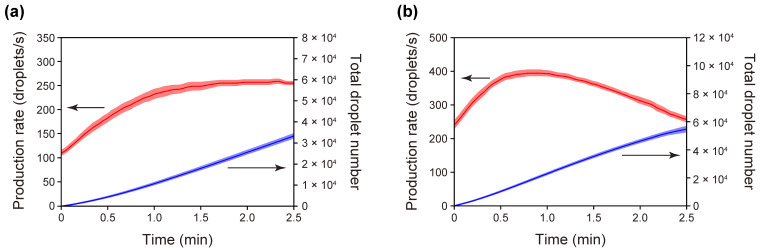
Changes in the rate of droplet production over the time-course of the experiments (*n* = 5) for (**a**) the Variant II microchip and (**b**) the Variant III microchip. In each of the graphs, the red line shows the mean droplet production rate, while the blue line shows the mean total number of droplets produced, which was calculated by integrating the production rate with respect to time. The colored shaded areas above and below the lines represent ±1 standard error of the mean.

We next changed the planar dimensions of the standard PMMA/PDMS microchip and performed droplet-production experiments using this modified PMMA/PDMS microchip (Variant II; refer to Table 1 and Figure S2). Because the Variant II microchip is identical to the standard microchip except for the doubled planar dimensions (i.e., doubled PDMS solid volume), a comparison of the droplet-production rates of the standard and Variant II revealed a dependence of the solid volume of the degassed PDMS on the attainable vacuum level, and thus on the fluid-flow rates and the droplet-production rate. Figure 6a shows the changes in the droplet-production rate over time for the 5 repeated runs with the Variant II microchip. Droplets with a diameter of 86 µm were produced at a rate as high as ca. 260 droplets/s, with a total volumetric-flow rate of 199 nL/s, which was comparable to that achieved with the standard microchip (ca. 270 droplets/s with a total volumetric-flow rate of 194 nL/s). It is thus conceivable that an increase in the solid volume of the degassed PDMS slab did not increase the vacuum level inside the confined outlet space of the microchip.

Our previous study suggested that the vacuum level attained with our droplet production microchips is likely to be determined by the PDMS SA/V ratio within the air-trapping void space [50]. Therefore, a simple approach for increasing the droplet-production rate with a fixed PDMS SA/V ratio is to decrease the hydrodynamic resistances of the microfluidic channels. We halved only the lengths of the T-junction microchannels of the standard microchip and left all other structural conditions unchanged (Variant III; refer to Table 1 and Figure S2). As we expected, the production rate of droplets (diameter of 75 µm) for the Variant III microchip (~390 droplets/s with a total volumetric-flow rate of 236 nL/s) was clearly higher than that for the standard microchip (~270 droplets/s with a total volumetric-flow rate of 194 nL/s) (Figure 6b).

## 4. Conclusions

We instantly created microchannels of an intended pattern on a PDMS slab by directly engraving grooves on the surfaces of the slab through CO_2_-laser irradiation [36]. Another PDMS slab (as a top layer) placed on the microfluidic PDMS slab (as a bottom layer) reversibly sealed the laser-engraved microchannels, owing to the viscoelastic properties of PDMS. This PDMS/PDMS microchip allowed for the automatic production of monodispersed W/O droplets by simply degassing the microchip before injecting an oil phase and an aqueous phase into the defined inlet reservoirs. While PDMS is an affordable polymer material and is widely used in microfluidics studies, viscous liquid PDMS needs to be mixed with a curing agent, with the bubbles removed, and heating provided prior to being used as a solidified material. Such processes reduce the efficiency of manufacturing our connection-free droplet-production microchips. In the current study, we showed that the bottom PDMS slab can be replaced with a less expensive and more convenient ready-to-use polymer slab, such as a PMMA slab, without decreasing the droplet-production rate or the size uniformity of the formed droplets. In addition, the experimental results obtained in the current study indicate that the solid volume of the degassed PDMS slab does not affect the vacuum level attained within the microchip and thus the droplet-production rate. This finding suggests the feasibility of automatic droplet production with a reduced size of the PMMA (or other polymers)/degassed PDMS microchip at a rate comparable with that achieved via the current microchip format.

## Figures and Tables

**Table 1:** Comparison of the structural architecture of the previous and current microchips.

Microchip ID	Material for the Bottom slab ^a^	Dimensions of the Bottom slab (W × D × H)	Dimension of the Top PDMS slab (W × D × H) ^b^	Channel Length ^c^
Previous model [36]	PDMS	76 mm × 26 mm × 4 mm	76 mm × 26 mm × 4 mm	4 mm each
Standard (this work)	PMMA	76 mm × 26 mm × 2 mm	76 mm × 26 mm × 4 mm	4 mm each
Variant I (this work)	PET	76 mm × 26 mm × 2 mm	76 mm × 26 mm × 4 mm	4 mm each
Variant II (this work)	PMMA	76 mm × 52 mm × 2 mm	76 mm × 52 mm × 4 mm	4 mm each
Variant III (this work)	PMMA	76 mm × 26 mm × 2 mm	76 mm × 26 mm × 4 mm	2 mm each

^a^ PDMS, polydimethylsiloxane; PMMA, poly(methyl methacrylate); PET, poly(ethylene terephthalate). ^b^ For increasing the attainable vacuum level, a two-dimensional array of orthogonal microgrooves (covering an area of 850 mm^2^) was fabricated on the upper surface of each of the top PDMS slabs listed above. ^c^ Lengths of the 3 individual microchannels from the T-junction point to each of the channel terminals (i.e., R1, R2, and R3 in Figure 1 and Figure S2).

**Table 2:** Variation of droplet diameter for each experiment.

Run	*n* ^a^	Mean (µm)	SD (µm)	CV (%)
#1	3264	82	2.9	3.5
#2	3439	85	3.6	4.2
#3	2380	86	1.6	1.9
#4	4533	84	1.7	2.0
#5	3254	82	3.4	4.1

^a^ Total droplet sample size. SD, standard deviation; CV, coefficient of variation. Note that the microchip was degassed before each run.

## Supplementary Materials

The following supporting information can be downloaded at: https://www.mdpi.com/article/10.3390/mi13091389/s1, Figure S1: Schematic representation of the operational steps in the autonomous pumping process; Figure S2: Schematic representation of the microchips used in the preceding work (a) and the current work (b−e).
