# Supplementary material for: CO2-Laser-Micromachined, Polymer Microchannels with a Degassed PDMS slab for the Automatic Production of Monodispersed Water-in-Oil Droplets
Source: Micromachines (Basel). 2022 Aug 25;13(9):1389. doi: 10.3390/mi13091389 (PMC9502940; doi:10.3390/mi13091389)
Supplement: Supplementary file 1 [file micromachines-13-01389-s001.zip › micromachines-1786753-supplementary.pdf]

CO_2-Laser-Micromachined, Polymer Microchannels with a Degassed PDMS slab for the Automatic Production of Monodispersed Water-in-Oil Droplets

Akitsu Ogo, Shotaro Okayama, Masaya Nakatani and Masahiko Hashimoto *

Department of Chemical Engineering and Materials Science, Faculty of Science and Engineering, Doshisha University, 1-3 Tataramiyakodani, Kyotanabe, Kyoto 610-0321, Japan

* Correspondence: mahashim@mail.doshisha.ac.jp

Table of Contents

1 Principle of the Autonomic Pumping Method

2 Comparison of structural architecture of microchips used in the preceding and current studies

1 Principle of the Autonomic Pumping Method

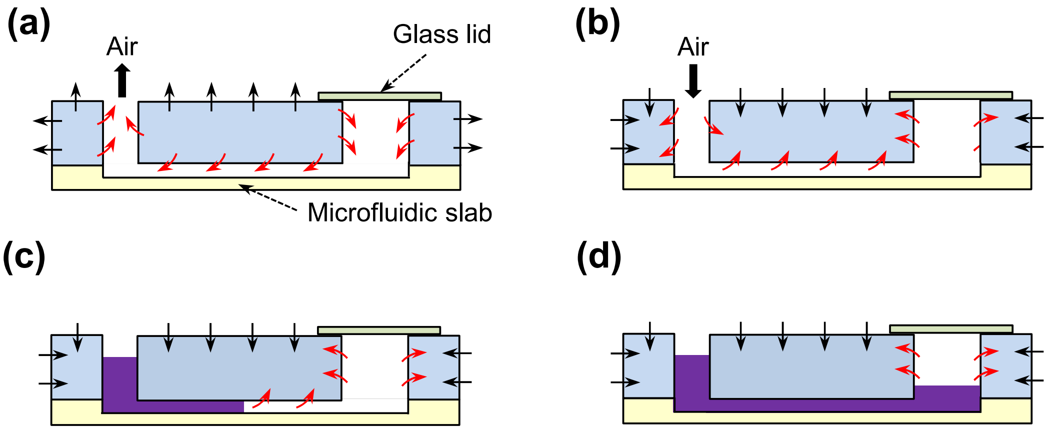

Figure S1. Schematic representation of the operational steps in the autonomous pumping process. The light blue rectangles represent PDMS, while the arrows show air-flow directions. Among the arrows shown in the figures, red ones indicate air-flow directions in the reservoirs and the channel.

As shown in Figure S1a, air dissolved in the vicinity of the channel wall is evacuated through the channel and the inlet reservoir (left one). Air cannot go through the outlet reservoir (right one) because the open-top of the reservoir is sealed with a lid (cover slip or adhesive tape). When the PDMS device is put back to atmospheric pressure environment, the directions of air transfer are reversed (Figure S1b). When a liquid is dispensed into the inlet reservoir, dissolution of air into PDMS starts to reduce the pressure inside the channel and the outlet reservoir because air is no longer fed from the outside. As a consequence, the liquid in the inlet reservoir begins to flow in the channel autonomically (Figure S1c). The liquid flow lasts even after the entire channel was filled with the liquid because the remaining air confined in the outlet still permeates into the PDMS (Figure S1d).

2 Comparison of the structural architecture of microchips used in the preceding and current studies

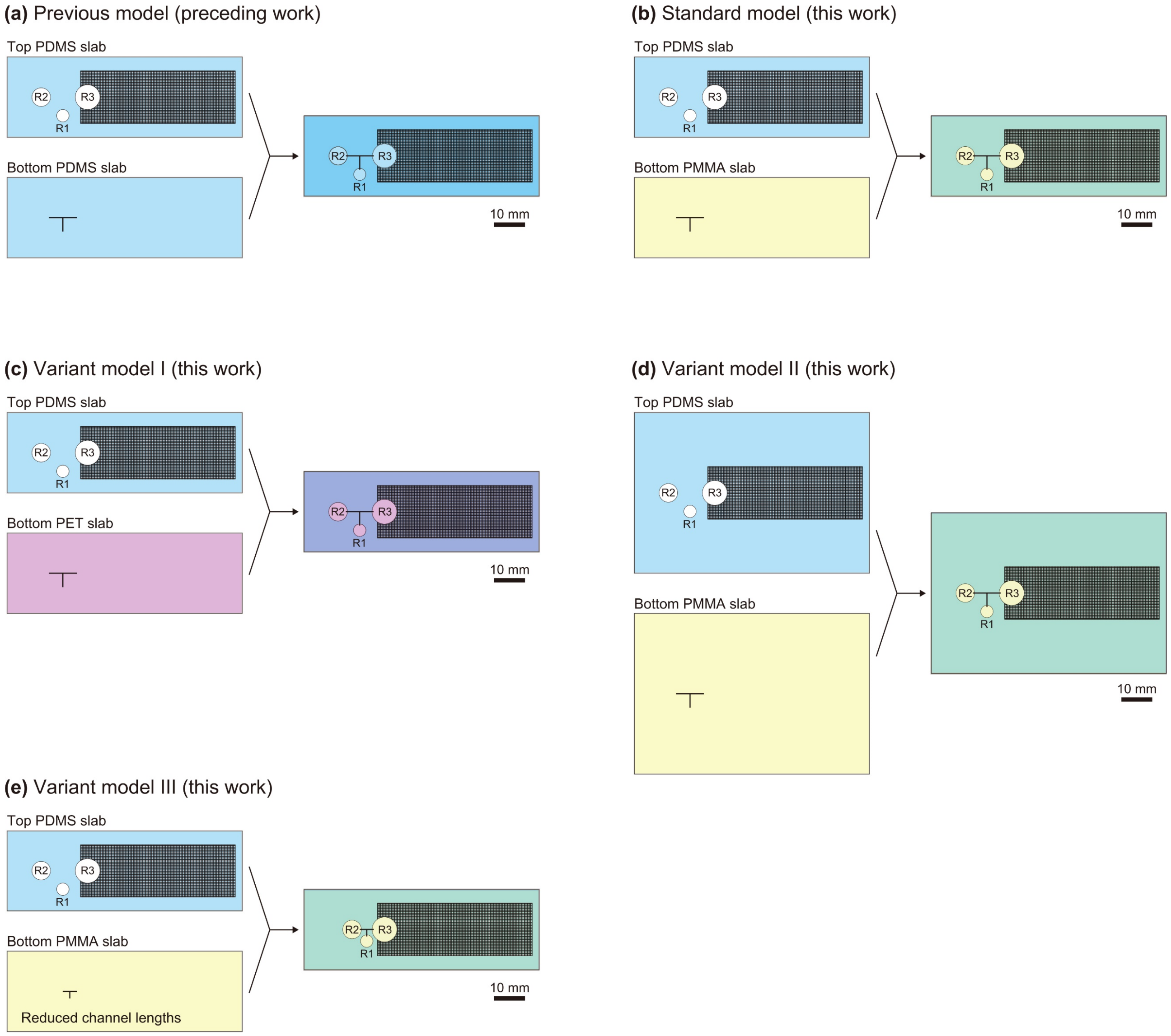

Figure S2. Schematic representation of the microchips used in the preceding work (a) and the current work (b−e).
